# Dynamic Tissue Rearrangements during Vertebrate Eye Morphogenesis: Insights from Fish Models

**DOI:** 10.3390/jdb6010004

**Published:** 2018-02-28

**Authors:** Florencia Cavodeassi

**Affiliations:** Institute of Medical and Biomedical Education, St George’s, University of London, London SW17 0RE, UK; fcavodea@sgul.ac.uk

**Keywords:** live-imaging, eye, morphogenesis

## Abstract

Over the last thirty years, fish models, such as the zebrafish and medaka, have become essential to pursue developmental studies and model human disease. Community efforts have led to the generation of wide collections of mutants, a complete sequence of their genomes, and the development of sophisticated genetic tools, enabling the manipulation of gene activity and labelling and tracking of specific groups of cells during embryonic development. When combined with the accessibility and optical clarity of fish embryos, these approaches have made of them an unbeatable model to monitor developmental processes in vivo and in real time. Over the last few years, live-imaging studies in fish have provided fascinating insights into tissue morphogenesis and organogenesis. This review will illustrate the advantages of fish models to pursue morphogenetic studies by highlighting the findings that, in the last decade, have transformed our understanding of eye morphogenesis.

## 1. Introduction

Organogenesis is a highly orchestrated process that involves the coordination of cell fate specification and the morphogenesis of differentiating tissues to give rise to the final architecture of mature organs. Tissue morphogenesis involves cell proliferation, changes in cell shape and cell polarity, and precisely coordinated cell movements. As they reorganise, cells in a tissue influence their surroundings, and by exerting forces and tension on neighbouring cells they contribute to the formation of a functional, mature organ. The recent development of new labelling and imaging techniques has allowed for starting to record, in real time and with cellular and subcellular resolution, the cell dynamics that accompany tissue morphogenesis (reviewed in [1,2]). Tools have been developed to measure forces in developing tissues, and to manipulate molecular and mechanical cell properties, and assess their impact on morphogenesis [3,4]. Tropical fish, such as zebrafish and medaka, are amongst the most popular model organisms to pursue this type of analysis. Indeed, the small, transparent fish embryos allow detailed live imaging of any morphogenetic process, even those involving cell rearrangements deep in the embryo. When combined with the availability of transgenic tools to label specific tissues, cellular and subcellular structures, and the extensive array of embryological and genetic manipulations that can be performed in fish, the last few years have witnessed an explosion of imaging studies that provide detailed analysis of many organogenesis events (see for example [5,6,7,8]). The power of live imaging to uncover cellular mechanisms driving tissue morphogenesis is best exemplified by the studies that, during the last 10 years, have transformed our understanding of eye morphogenesis. This review will focus on these studies and will provide a reflection of where these findings are taking us next.

## 2. Overview of Eye Morphogenesis

**Figure 1:**
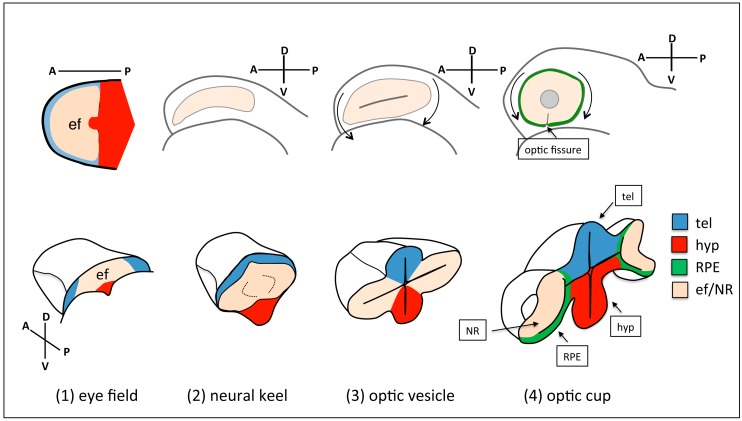
Schematic of eye morphogenesis in zebrafish. Eye field specification in the anterior portion of the neural plate (ANP) (**1**) is followed by the rearrangement of the tissue as the neural plate folds into a keel (**2**), and the evagination of the optic vesicles (**3**). Subsequent folding of the optic vesicle over itself leads to the formation of the optic cup (**4**). Top-left panel is a dorsal view with anterior to the left; the orientation of all the other panels is indicated in the figure. tel: telencephalon; hyp: hypothalamus; RPE: retinal pigment epithelium; ef: eye field; NR: neural retina.

The eye primordium is specified in the anterior portion of the neural plate (ANP) as a single domain spanning the midline. As the neural plate folds to give rise to the neural tube, the eye field cells are displaced laterally and evaginate to give rise to the optic vesicles, the first morphological manifestation of the eyes in the embryo [9,10]. Optic vesicle evagination is followed by the asymmetric folding of the distal portion of the eye primordium to give rise to the optic cup, which is a bilayered structure where the external layer will become the retinal pigment epithelium (RPE) and the internal layer will become the neural retina (NR). The asymmetric folding of the cup leaves a gap along the ventral portion of the optic primordium known as the choroid (or optic) fissure, which at late stages of eye maturation, will close and fuse to give rise to continuous NR and RPE layers. Throughout this whole process the distal eye structures are physically connected to the brain by the optic stalk. The optic stalk will eventually remodel to give rise to the cell types that ensheathe the optic nerve [11]. Further maturation of the NR involves the proliferation and differentiation of retinal progenitors to give rise to all the neuronal and glial cell types that compose the retina [12,13]. RPE cells, instead, undergo marked changes in cell morphology and eventually differentiate to give rise to a squamous, pigmented epithelium that covers the whole apical surface of the retina and preserves photoreceptors function [14,15] (Figure 1). The extensive reshaping of the eye primordium as development proceeds is highly conserved across vertebrates and can be reproduced in vitro in organoid models [16,17], emphasising the highly self-regulative properties of this process. It is likely that changes in individual cells’ shape and organisation will somehow drive the remodelling of the eye at a tissue level; however, to understand the impact of cellular and subcellular processes on eye morphogenesis, it is necessary to capture the dynamics of changes in cell shape and organisation in real time. Live imaging of eye development has been performed in detail in fish models, and has allowed documenting cell behaviours from the earliest dynamic reorganisations of eye field cells, to the detailed differentiation paths taken by retinal progenitors in the mature optic cup.

## 3. Visualising Global Cellular Rearrangements and Tissue Remodelling

The specification of the eye precursors occurs concomitant to the subdivision of the ANP in several domains that will give rise to the different parts of the brain. Once specified, eye progenitors follow a distinctive morphogenetic program. By labelling and recording the organisation of ANP cells during the narrowing and initial folding of the neural plate, England and colleagues [18] showed that eye cells initially condense along the anterior-posterior (AP) axis, concomitant with the anterior movement of future hypothalamic cells. Subsequently, eye field cells at the ventral midline are displaced anteriorly and outwards by the anterior relocation of future dorsal diencephalic cells. As this occurs, more dorsally located eye field cells are also laterally displaced by the dorsal convergence and closure of the future telencephalon. The reorganisation of the ANP, and in particular of the eye field, was also documented by Rembold and colleagues [19], using the medaka fish as a model, and a transgenic line (*Tg{rx3::GFP}*) that specifically labels eye field cells. They further showed that the evagination of the eye field from the lateral walls of the neural keel to give rise to the two optic vesicles is a highly dynamic process reminiscent of active cell migration. These studies highlighted the active reorganisation that eye cells undergo during optic vesicle formation in relation to surrounding tissues, and how tightly coordinated eye and forebrain morphogenesis are. They also led to new hypotheses around the molecular mechanisms driving eye field cell rearrangements. For example, the Wnt molecule Wnt11 is required locally in the eye field to maintain the cohesion of this domain [20], but it also indirectly affects the ability of eye cells to move laterally prior to evagination, due to its role in driving neural plate narrowing and anterior migration of the prechordal plate [18]. The subsequent lateral expansion of the optic vesicles required the function of the eye-specification gene *rx3*, such that in the absence of Rx3 function eyeless embryos develop [19,21]). The analysis by Rembold and colleagues, extended later by Brown and colleagues [22] suggested that this is due to an inability of eye cells devoid of Rx3 to move away from the midline, behaving instead similarly to telencephalic cells.

As the optic vesicle matures and transforms into an optic cup, further remodelling of the tissue occurs. Global analysis of this whole process was performed by Kwan and colleagues [23], who generated over 12 hour movies spanning from the last stages of optic vesicle evagination to optic cup stages. By retrospectively tracking cells allocated in different optic cup regions at the end of the movie, they were able to determine the initial position of future RPE and NR cells in the optic vesicle. This study, together with others [23,24,25], also showed that as the optic vesicle folds to give rise to the optic cup, the cells in the primordium reorganise extensively; future RPE cells spread throughout the external layer of the optic cup, while a subset of NR cells initially positioned in the external layer of the primordium relocate to the internal layer to acquire their final position in the optic cup. These cell rearrangements may provide some of the driving force that is required for the efficient folding of the eye primordium.

All of these studies made use of conventional confocal microscopy and a whole repertoire of transgenic lines and other labelling approaches to document cell movements and tissue rearrangements, but early eye morphogenesis has also been documented by light-sheet microscopy, which is an advanced imaging technique that allows for the analysis of the whole cohort of cells in the embryo [26,27]. These studies, although not specifically directed to analysing eye morphogenesis, have provided movie sets with a high level of cellular resolution allowing the reconstruction of cellular paths over extended developmental times.

## 4. Following Changes in Cell Shape and Cytoskeletal Dynamics

**Figure 2:**
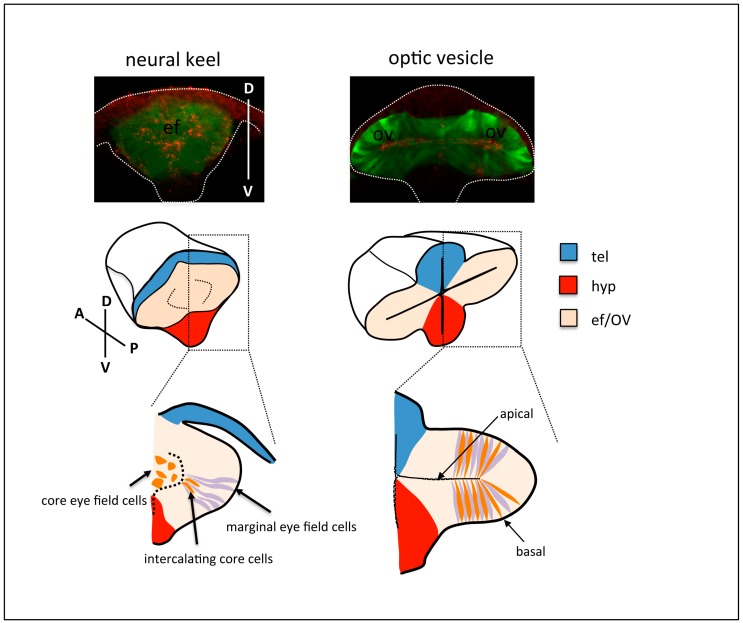
Top row: frontal views of neural keel (**left**) and optic vesicle (**right**) stage *Tg{rx3::GFP}* zebrafish embryos, immunostained to highlight the eye field/optic vesicles (GFP expression, green) and the apical domain of the tissue (ZO-1, red). The contour of the embryos is outlined. Middle and bottom row: schematic of cell rearrangements during optic vesicle evagination. Cells at the margin of the eye field (purple) organise as a columnar epithelium at the onset of optic vesicle evagination. Subsequent expansion of the optic vesicles is accompanied by the gradual intercalation and elongation of cells in the core of the eye field (orange) amongst marginal cells. tel: telencephalon; hyp: hypothalamus; ef: eye field; OV: optic vesicle.

The studies above analysed global cell dynamics, but did not address the structure of the tissue at a cellular level, nor the shape of the cells and the organisation of their cytoskeleton and junctional complexes. More recent studies revisited eye morphogenesis to provide a detailed understanding of the changes in cell shape and cell polarity that drive optic vesicle evagination and folding. Ivanovitch and colleagues [28] re-analysed the transition from eye field to optic vesicle, in order to uncover the organisation of eye cells within the tissue during these stages. By imaging eye morphogenesis from a transverse point of view and labelling cell contours and apical compartments, Ivanovitch et al. found that a subset of eye field cells at the margin of the neural keel are highly organised as a columnar epithelium from the onset of evagination. Following in detail individual cell shape and polarity revealed that this columnar epithelium thickens as neuroepithelial cells elongate, and becomes expanded mediolaterally by the gradual intercalation, elongation, and polarisation of eye cells at the core of the eye field domain (Figure 2). The net result of these cellular rearrangements is the lateral evagination and expansion of the eye field to give rise to the optic vesicles. Their study further showed that the initial trigger for the columnar organisation and polarisation of eye cells was the local assembly of a Laminin-rich basement membrane around the eye field; in the absence of Laminin function, eye cells fail to organise and elongate, and optic vesicle evagination is severely disrupted; conversely, an exogenous source of Laminin placed in the centre of the eye field instructs polarity around itself and distorts the organisation of eye field cells, thus generating ectopic lumens within the eye primordia.

**Figure 3:**
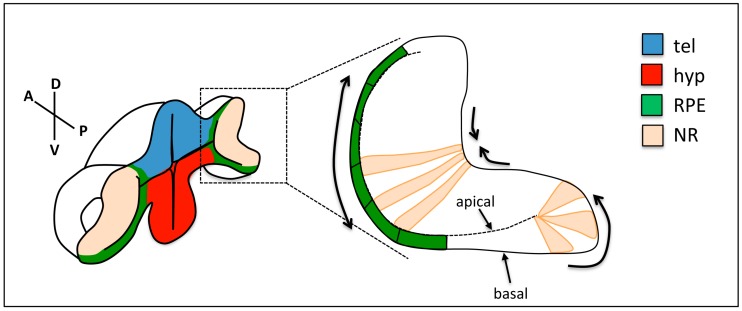
Changes in cell shape during optic cup folding. Prospective retinal pigment epithelium (RPE) cells (green) flatten, cells at the rim show apical constriction and basal lamellipodia, and neural retina (NR) cells show basal constriction. Arrows show the expected direction of tissue movement. tel: telencephalon; hyp: hypothalamus; RPE: retinal pigment epithelium; NR: neural retina.

Subcellular labelling of cytoskeletal components was also exploited to analyse the mechanisms driving subsequent stages of eye morphogenesis. As described above, the invagination of the optic vesicle to give rise to the bilayered optic cup is accompanied by a flow of cells around the rim of the primordium and their relocation from the external to the internal layer of the optic cup. Even though the driving force for this dynamic reorganisation is currently unclear, marked changes in cell shape have been documented during this process (Figure 3). A small subset of cells in the proximal region of the external layer of the optic vesicle (future RPE cells [23,29]) become shorter in the apico-basal (AB) axis and expand their surface, potentially displacing surrounding cells towards more distal regions as they do so. As prospective NR cells in the external layer approach the rim of the primordium, they elongate and undergo a marked apical constriction, while at their basal side, they display active lamellipodial activity. The net result is the displacement of externally located NR cells around the rim, and their relocation to the internal layer of the optic primordium [30]. Concomitant to these cell shape changes in the external layer of the optic cup, future NR cells in the internal layer of the invaginating primordium elongate in the AB axis and compact in the plane of the epithelium [24]. In addition, their basal surfaces constrict, while maintaining a wider apical surface, so that the global effect is the bending of the basal side of the NR [31,32,33].

Optic cup folding generates a fissure along the ventral portion of the primordium, and during late stages of optic cup formation, the lips of the optic fissure fuse to give rise to a continuous retina and RPE. This poorly understood process involves the fusion of two epithelia along their basal sides. For this fusion to occur, the basement membrane that covers them needs to be broken down, and cell rearrangements need to occur that align NR and RPE at both sides of the fissure (reviewed in [11]). Recent imaging studies have started to unravel the dynamics of these processes. Indeed, basement membrane breakdown precedes optic fissure fusion, and persistent basement membrane interferes with fusion [34]. Tethering of both sides of the optic fissure to each other, basement membrane breakdown, and eventually fissure closure requires the migration of a population of periocular mesenchymal (POM) cells into the fissure and the formation of the hyaloid vasculature [34,35,36,37], highlighting the dynamic interaction between the different tissues that are required for late stages of optic cup morphogenesis.

Ultimately, the changes in cell shape described during optic cup folding and choroid fissure closure are likely driven by the dynamic reorganisation of the cells’ cytoskeleton and adhesion complexes. For example, NR cells basal constriction involves the stabilisation of basal localisation of focal adhesion complexes, which is a process that is controlled by ojoplano (opo), a conserved protein that localises to the basal end feet of NR cells, where it cooperates with the clathrin adaptors Numb and Numbl to regulate integrin endocytosis [31,32]. Analysis of the distribution of cytoskeletal components during optic cup folding revealed an enrichment of actomyosin at the basal surface of NR cells [30,33]. Recording actomyosin dynamics further revealed that this enriched cytoskeletal network promotes pulsatile behaviour of NR cell membranes, ultimately leading to the constriction of the basal surface of the NR [33]. Indeed, pulsatile behaviour and gradual constriction is associated to the episodic formation of foci of myosin accumulation, which seem to pull from the underlying ECM. Thus, the cell intrinsic gradual constriction of NR basal end feet is likely translated into tension at the tissue level by the linkage of the cells’ cytoskeleton to the ECM through focal adhesions. Indeed, interfering with either the contraction of the cortical cytoskeleton, the assembly of focal adhesions or the assembly of the ECM eventually results in a release of tissue tension and failure of the NR to bend [31,32,33,38]. The changes in cell shape driving rim cell involution are also controlled by the cell cytoskeleton, which is remodelled at the basal surface leading to the extension of dynamic actin-rich protrusions in the direction of movement. Interference with rim cell involution eventually leads to defects in optic cup folding, and to the ectopic location of NR cells in the external layer of the optic cup [24,25,30].

The studies discussed above have identified a number of cellular behaviours (flattening of the prospective RPE cells, basal constriction of NR cells, active collective cell migration at the optic cup rim; active migration of the POM cells into the optic fissure) that are likely to contribute to the efficient folding of the optic cup and to the accurate positioning of NR and RPE cells into the eye primordium. However, it is still unclear from these studies whether there is a functional coupling between these different mechanisms, or whether they act independently and cooperatively to shape the optic cup. In the absence of Opo activity, the optic cup does not fold efficiently, and NR cells end up ectopically positioned in the external layer of the primordium, suggesting that interference with NR basal cells constriction may impact on rim cell involution [33]. Indeed, the tension generated by NR cell compaction and basal constriction may be enough to “pull” from cells at the rim, generating the force driving rim cell involution. Similarly, the flattening of RPE cells and their expansion in the surface of that tissue may “push” surrounding cells towards the rim promoting their relocation to the internal layer. However, determining the exact contribution of each tissue to the morphogenesis of the optic cup is not trivial, since the same molecular machinery seems to control all these cell behaviours. The development of tools to locally interfere with cytoskeleton dynamics in each of these optic cup regions will be crucial to resolve these issues.

## 5. Assessing the Coordination between Fate Specification and Morphogenesis

The shaping of the eye primordium occurs simultaneously to the gradual restriction in the fate of discrete groups of eye cells. Indeed, as the optic vesicle evaginates, it simultaneously becomes patterned to give rise to a proximal, future optic stalk domain, and distal RPE/NR domains (reviewed in [10,39]). In addition, axial patterning of the prospective NR starts to be established at the onset of optic vesicle evagination. Future nasal and temporal domains can already be visualised by the expression of specific nasal/temporal fate markers from the earliest stages of optic vesicle evagination. Studies in zebrafish have shown that signals from the Fibroblast-growth-factor (Fgf) and Hedgehog (Hh) families are required to establish these domains [24,40]. Optic vesicle evagination involves extensive cell rearrangements, and thus to have a full picture of how the architecture of the tissue is generated, one needs to be able to monitor cell fates as they are established in vivo and relate this to the morphogenetic rearrangement of the differentiating tissue. Crucial for this is the availability of tools that allow for us to visualise signalling pathway activation and fate establishment in vivo. In recent years many tools of this type have been developed [41,42,43,44,45,46,47,48], but they have not been applied to this purpose during eye morphogenesis.

The establishment of nasal/temporal patterning during optic vesicle evagination nicely exemplifies how this type of tools might help us understand the tight coordination of morphogenesis and fate acquisition. As already discussed, eye field cells at the margin of the neural keel are organised as a columnar epithelium prior to optic vesicle evagination. As optic vesicle evagination proceeds, this columnar epithelium thickens and becomes expanded mediolaterally by the subsequent intercalation, elongation and polarisation of the remaining eye cells, which were initially located at the core of the eye field domain [28]. This process of cell intercalation is apparently random, since intercalating cells populate all the regions of the evaginating optic vesicles. Dorsal (future nasal) and ventral (future temporal) domains of differential gene expression can already be detected from the onset of evagination, prefiguring the nasal/temporal pattern of the eye primordium [24]. Thus, intercalation of core eye cells occurs in an already patterned primordium, and as they integrate in either the dorsal or the ventral domain, they acquire nasal or temporal fates. How this process is coordinated is currently unclear. Do core cells intercalate randomly and subsequently acquire the corresponding cell fate, or instead, their intercalation into the dorsal or ventral domain is driven by their predetermination to one of these two fates? Recent studies showing that nasal and temporal fates are established by the coordinated action of Fgf and Shh signals [24,40] provide the necessary background knowledge to answer these questions. Indeed, the availability of nasal and temporal fate markers [24,40], and the existence of Shh and Fgf signalling pathway reporter lines [46,47], provides an entry point to assess how signals are received and interpreted by cells that are constantly changing their position. The design and application of similar tools to other stages of eye morphogenesis will allow us in the long term to gain a complete picture of the early events leading to the shaping and patterning of the eye primordium.

## 6. Perspective

The studies discussed above have provided us with a detailed understanding of the changes in cell shape and organisation that are involved in the shaping of the eye primordium in fish models. They have also allowed for us to start grasping the mechanical consequences of such changes at a cellular level, and to start formulating hypotheses on the mechanisms driving eye morphogenesis. Nevertheless, it is still unclear up to what extent these mechanisms are conserved in other organisms, particularly in mammals. One crucial limitation is the fact that a similar dynamic analysis of eye morphogenesis has not been conducted in mammals, and all of the available information comes from static analysis of fixed tissues. In addition, the structure and organisation of the embryonic tissues at the onset of eye morphogenesis in mammals is different to that described for fish embryogenesis. For example, while in the zebrafish the neural plate is not yet organised as a mature neuroepithelium at the onset of eye evagination, in mammals, this is indeed the case. However, many similarities do also exist. Indeed, a similar sequence of cell elongation at the onset of optic vesicle evagination has been suggested from the analysis of sectioned mouse tissue [49]. Moreover, recent experiments inducing in vitro optic cup formation in three-dimensional (3D) cultures of mouse and human embryonic stem cells have shown that the successful morphogenesis of eye organiods requires, as in zebrafish, the presence of a Laminin-rich extracellular matrix [16,17]. Subsequent folding of the optic cup progresses in similar ways in fish models and mammals, and the final bilayered structure of the optic cup is mostly identical in all vertebrates. In mammals, the RPE has been proposed to provide a rigid scaffold that promotes the folding of the optic cup [50,51], and marked changes in cell shape have been documented at the interface between the RPE and NR during optic cup folding in eye organiods [16,17]. Thus, the mechanistic differences that may exist early on likely reflects the fact that in fish models, early development occurs at a very fast pace, and the specification of tissues, their assembly and initial spatial reorganisation extensively overlap in time.

Not only global eye tissue rearrangements are largely comparable across species, but also the gene regulatory network (GRN) that is involved in triggering fate specification and regional patterning during eye development is mostly conserved [15,52,53,54]. Moreover, many of the genes and signalling pathways that are crucial for eye specification, morphogenesis, and patterning in fish are affected in human ocular malformations (reviewed in [55]; some examples in [56,57,58,59]). We are however still far from understanding how the GRN involved in controlling eye patterning ultimately controls cell behaviours and tissue morphogenesis, and how the emerging biomechanical properties of the differentiating optic cup domains impacts on organ shape. We can be certain that the zebrafish will make a crucial contribution to these open questions in the years to come.
